# Rabies in Kazakhstan

**DOI:** 10.1371/journal.pntd.0004889

**Published:** 2016-08-03

**Authors:** Akmetzhan A. Sultanov, Sarsenbay K. Abdrakhmanov, Aida M. Abdybekova, Bolat S. Karatayev, Paul R. Torgerson

**Affiliations:** 1 Kazakh Research - Scientific Veterinary Institute LLP, Almaty, Kazakhstan; 2 Kazakh Agrotechnical University named after S. Seifullin JSC, Astana, Kazakhstan; 3 Section of Veterinary Epidemiology, University of Zürich, Zürich, Switzerland; Swiss Tropical and Public Health Institute, SWITZERLAND

## Abstract

**Background:**

Rabies is a neglected zoonotic disease. There is a sparsity of data on this disease with regard to the incidence of human and animal disease in many low and middle income countries. Furthermore, rabies results in a large economic impact and a high human burden of disease. Kazakhstan is a large landlocked middle income country that gained independence from the Soviet Union in 1991 and is endemic for rabies.

**Methodology/Principal Findings:**

We used detailed public health and veterinary surveillance data from 2003 to 2015 to map where livestock rabies is occurring. We also estimate the economic impact and human burden of rabies. Livestock and canine rabies occurred over most of Kazakhstan, but there were regional variations in disease distribution. There were a mean of 7.1 officially recorded human fatalities due to rabies per year resulting in approximately 457 Disability Adjusted Life Years (DALYs). A mean of 64,289 individuals per annum underwent post exposure prophylaxis (PEP) which may have resulted in an additional 1140 DALYs annually. PEP is preventing at least 118 cases of human rabies each year or possibly as many as 1184 at an estimated cost of $1193 or $119 per DALY averted respectively. The estimated economic impact of rabies in Kazakhstan is $20.9 million per annum, with nearly half of this cost being attributed to the cost of PEP and the loss of income whilst being treated. A further $5.4 million per annum was estimated to be the life time loss of income for fatal cases. Animal vaccination programmes and animal control programmes also contributed substantially to the economic losses. The direct costs due to rabies fatalities of agricultural animals was relatively low.

**Conclusions/Significance:**

This study demonstrates that in Kazakhstan there is a substantial economic cost and health impact of rabies. These costs could be reduced by modifying the vaccination programme that is now practised. The study also fills some data gaps on the epidemiology and economic effects of rabies in respect to Kazakhstan.

## Author Summary

Kazakhstan is a large central Asian country that was part of the Soviet Union until 1991. The country is endemic for rabies. This study shows that there are areas of Kazakhstan such as the north and south east of the country where outbreaks of animal rabies are concentrated. Cattle, dogs and foxes are the animals most frequently confirmed with rabies. A mean of 7.1 human deaths annually due to rabies occurred between 2009 and 2015 inclusive in Kazakhstan resulting in 457 disability adjusted life years. A mean of 64,801 people each year are recorded as suffering bite injuries from animals, mainly due to dogs. Children are at higher risk of being bitten. However, the widespread use of post exposure prophylaxis (PEP) prevents at least 118 and possibly up to 1184 fatalities per annum of people bitten by rabid animals. The economic costs of this disease are high: exceeding $20 million per annum. However, the widespread use of PEP is cost effective in reducing the burden of disease.

## Introduction

Rabies is a fatal viral zoonotic disease largely transmitted to humans from bites by infected animals. Rabies is currently registered in over 100 countries in all continents except Australia and Antarctica. The disease is characterized by neuroencephalitis with 100% case fatality ratio once clinical signs present. Over 55,000 people and over 1 million animals die from rabies annually throughout the world. Direct losses from rabies amount to over EURO 4 billion each year [1].

Human rabies presents a serious public health threat in Kazakhstan. It is a reportable disease and public heath data is available to quantify numbers of cases. Kazakhstan is an upper middle income country. Previous published data suggested that between 2007 and 2011, 44 cases of human rabies were recorded or a mean of 9 cases per year. Of these 40 were the result of contact with dogs, 3 cases from contact with cats and 1 case from a fox. The incidence of dog bites was reported as 3700 per million population in 2010 and 4130 per million population in 2011. Post exposure prophylaxis was given to 57,000 individuals in 2009, 59,000 in 2010 and 67,000 in 2011 [2].

Like many endemic countries, animal rabies is registered annually within Kazakhstan. There is also a programme of vaccination of domestic dogs, cats and agricultural animals and a limited programme to vaccinate foxes through the distribution of vaccine impregnated baits. However a reduction in the disease incidence in animals has not been observed. Thus, one of the objectives of this study was to better understand the epidemiology of rabies by examining the geographical distribution of rabies amongst animals in the country and to evaluate control measures[3].

The economic and disease burden of rabies has also not been previously estimated specifically for Kazakhstan, although estimates, based on modelling, are available from the global burden of rabies study (GBR) [1]. Surveillance data on the number of cases of human rabies, the incidence of animal bites and the numbers of individuals undergoing post exposure prophylaxis (PEP) were used to obtain more precise estimates of the burden of disease. In addition, data from the number of bites received from confirmed rabid animals was used to estimate the burden of disease avoided by the use of PEP and its cost effectiveness. Finally the economic effects of the disease was estimated from the costs of the animal vaccination programme, the costs of control of potentially rabid animals, the costs of PEP, the loss of income through premature death and the cost of livestock lost to rabies.

## Materials and Methods

### Rabies diagnosis

Suspect animal rabies cases were examined at regional branches of the national veterinary laboratory services. To confirm the diagnosis of rabies, first the brain was removed. Samples were taken from the hippocampus and the cerebellar cortex for further examination. Tissue was examined using the direct fluorescent antibody test or for PCR using the VeTek RV Detection kit (iNtRON Biotechnology, Inc, Jungang Induspia V, Sangdaewon-Dong, Joongwon-Gu, Seongnam, Gyeonggi-Do, 462–120 KOREA; www.intronbio.com). In suspect cases where these tests proved negative bioassays were undertaken in mice. Briefly, material was homogenized, centrifuged and the supernatant inoculated intra cerebrally into mice. The mice were observed for up to 30 days. Rabies was confirmed by histology of brain samples from the mice showing negri bodies and/or Babes nodules consisting of glial cells. All confirmed cases of animal rabies were recorded in terms of animal species and exact geographical coordinates of the origin of the animal.

### Animal data

Data was extracted from diagnostic results provided by regional branches of the Republican Veterinary Laboratory and statistical data from the Ministry of Agriculture, Veterinary Control and Monitoring Committee reports. This data included the numbers of animals which were confirmed as rabies according to species. The GIS coordinates of every confirmed animal rabies cases was recorded from 2003 to 2013. An outbreak of rabies was defined as 2 or more cases found at the same time with the same GIS coordinates. The numbers of animals vaccinated against rabies was also reported and the expenditures associated with the capture and destruction of stray dogs for 2010–2015. The data used is provided in the supporting information S1 Dataset (compressed file archive). Total livestock populations were derived from statistical data of the Ministry of Agriculture.

### Human data

The number of human rabies cases was as reported by Republican Veterinary Laboratory and statistical data from the Ministry of Agriculture, Veterinary Control and Monitoring Committee reports from 2010 to 2015 and earlier from 2007 [2]. Likewise the numbers of individuals who suffered animal bites and were given post exposure prophylaxis (PEP) were also available from government statistics for this period. The population of Kazakhstan is reported as 17.1 million in 2010 rising to 18.2 million in 2015 [4].

### Vaccination programme

One of the mechanisms for disrupting transmission of rabies is the use of vaccination [5, 6]. Thus, in order to prevent rabies all 14 regions where the disease has been registered, annual regular vaccination of cattle, sheep and goats, horses, camels, dogs, and cats is undertaken. Data for the number of livestock and domestic pets vaccinated was recorded by the Ministry of Agriculture, Veterinary Control and Monitoring. Vaccines used were either Raksharab (Indian Immunologicals Ltd. Hyderabad, India) or Schelkovo-51 strain inactivated vaccine (Schelkovo Biocombinat, Moscow, Russia). Serum samples were obtained from 70 cattle and 30 sheep and goats vaccinated against rabies 3 months following vaccination to investigate antibody levels of vaccinated animals. Blood samples were taken and an indirect enzyme immunoassay for the quantitative determination of antibodies in sera was used (SERELISA Rabies Ab Mono Indirect, Synbiotics Europe (Lyon), France) to determine vaccine efficacy.

Control measures were evaluated based on changes in annual animal rabies occurrences. Starting in 2012, a vaccination programme of wild life was initiated using baits impregnated with live vaccine. To investigate the results of oral immunization in wild carnivores, tetracycline uptake from labelled vaccine baits was used [7].

The total number of doses of vaccine used by species and type of the vaccine depends on the total number of susceptible livestock. This is calculated based on the epidemiological situation specific to that district. For high risk areas, the present policy is to vaccinate all susceptible livestock and have a constant monitoring and vaccination of domestic and stray dogs and cats. It is also aimed to distribute vaccine widely to wildlife. In medium risk areas local vaccinations are undertaken in places where rabies had been registered in the last 3 years and monitoring of domestic and wild carnivores and local distribution of vaccine to wild life. In low risk areas where no rabies cases reported but thought to be possible, there is permanent monitoring of rabies in all species and control of domestic and wild carnivores. In very low risk areas where rabies is thought unlikely and is not recorded there is limited epidemiological screening and monitoring for evidence of rabies.

### Costs

Costs were estimated as the sum of the cost of animals vaccinations, funds allocated for the capture and destruction of stray and wild animals, livestock losses due to clinical rabies, the costs of PEP, losses of income whilst seeking treatment and of life time loss of income due to premature mortality.

The direct and indirect cost of PEP was not available in the data base and we were not able to obtain them from other sources. Therefore we used the data from GBR [1]. For Kazakhstan, this estimates the direct costs of PEP to be $1.95 million (CIs $0.99 -$4.3 million, travel costs for treatment, and additional $146,000 ($134,000-$566,000) and $5.55 million ($2.8million-$12.6 million) for lost income whilst seeking treatment. Using the uncertainty limits reported we fitted each of these cost items this to a betaPERT distribution using the R package “prevalence” [8]. The GBR study gave a mean estimate of 45620 (95% CIs 23,421 to 99,821)for the incidence of PEP treatment in Kazakhstan. This was also fitted to a betaPERT distribution. The we summed the total costs by taking random draws and dividing each by a random draw for the incidence of PEP. This was repeated 10,000 times and gave the estimated the cost per case of PEP as $147 (95% CIs $74-$258). This cost per case treated by PEP was therefore based on the incidence of PEP treatments estimated by the GBR study. However we then applied this cost to the higher incidence of PEP treatments in our data, again using a Monte-Carlo simulation. The cost of deaths from clinical rabies was the same as the capital approach used in the GBR study [1]. This method estimates the lifetime value of the lost income that would have accrued, from the age of death, were the individual not to have suffered from premature death. The mean annual income was estimated to be the annual gross domestic income per head. In 2014 this was reported by the World Bank to be US$11860 for Kazakhstan [9]

The values of animal production for cattle, small ruminants, horses and camels was taken from data from the FAO (available from http://faostat3.fao.org). The loss in animal production was estimated to be the proportion of animals lost to rabies multiplied by the value of animal production for each species. The costs of animal vaccination was the unit cost of vaccination multiplied by the numbers of animals vaccinated.

The costs allocated for the capture and destruction of stray and wild animals were directly available in the data bases and are provided in the supplementary information (S1 Dataset).

### Disability adjusted life years

These were estimated using standard techniques [10–13] without discounting or age weighting. The normative life table used for calculating years of life lost (YLLs) was based on the projected frontier life expectancy for 2050, with a life expectancy at birth of 92 years [13]. This is believed to be the average achievable life expectancy in the absence of disease or injury. Every country has a life expectancy at birth which is currently lower than this. The difference represents the mean YLLs lost due to injury or disease. As clinical rabies has a 100% case fatality ratio, YLLs were estimated from the numbers of reported cases of clinical human rabies. As only total human rabies cases were available and no breakdown according to age, the age of these cases was assumed to follow the same distribution as the age of bite victims. As a scenario a disability weight of 0.108 with a mean duration of 60 days was used to estimate YLDs for those patients who suffered bite injuries and underwent post exposure prophylaxis [1].

### Cases averted

The numbers of human rabies cases averted were estimated from the data on total number of bite injuries, the anatomical distribution of bite injuries and data on confirmed rabies cases of animals inflicting the bite injury. The probability of rabies occurring in an individual who is exposed to rabies through a bite from an infected animal is from [14]. Thus bites to the head have a probability of 0.55 (CIs 0.28–0.79) of transmitting rabies, bites to the upper extremity a probability of 0.22 (0.12–0.38), to the trunk 0.09 (0.05–0.38) and to the lower extremity 0.12 (0.06–0.23).

As an alternative scenario rather than using the proportion of bite injuries that were confirmed as being inflicted by an animal with rabies, we estimated the probability of being bitten by a rabid animal from the proportion of animals who were tested for rabies which subsequently were proven to be positive. This was because only a small proportion of animals that bit humans were available for rabies investigation and this could give an alternative estimate of the probability of an animal having rabies given that it has bitten a human.

In both scenarios we assumed that PEP, if given appropriately, is close to 100% effective in preventing rabies [15].

### Analysis

The geographical distribution of rabies cases was analysed by mapping the incident cases in animals onto relevant coordinates using Global Position System receivers (eTrex Legend, Global Sat GH-801 and Shturman SVG-40). The distribution and density of total rabies cases was analysed by kernel density [16] and displayed using the function smoothScatter in the R graphics package [17]. Calculations of economic losses and disease burden were also undertaken in R. All estimates incorporated uncertainty using Monte-Carlo simulations. Thus random draws from appropriate probability distributions were made. These were summed for DALY or cost estimates and repeated 10,000 times. Mean and 95% percentiles were then calculated from the results of the 10,000 simulations.

To explore the hypothesis that foxes may be the principal reservoir hosts, we also analysed the total number of cases in agricultural animals and humans by oblast in a generalized linear model (GLM) with numbers of confirmed cases in foxes and dogs as independent variables. Exploratory data analysis indicated that the mean number of cases per oblast (for every species) was much lower than the variance of the mean and, because the data is also left bounded by zero, a negative binomial regression model seemed to be the most appropriate statistical model for this analysis. We used the glm.nb function from the MASS package in R for this analysis.

The R code and data for all analyses is provided in the supplementary material (S1 Dataset).

## Results

**Fig 1:**
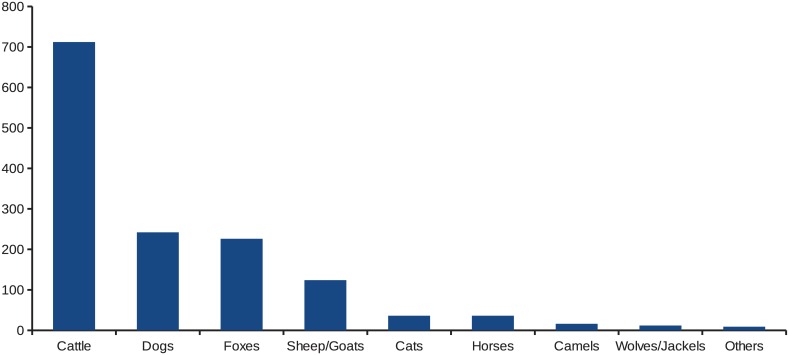
Numbers of confirmed rabies cases in animals by species affected between 2003 and 2015 in Kazakhstan.

**Fig 2:**
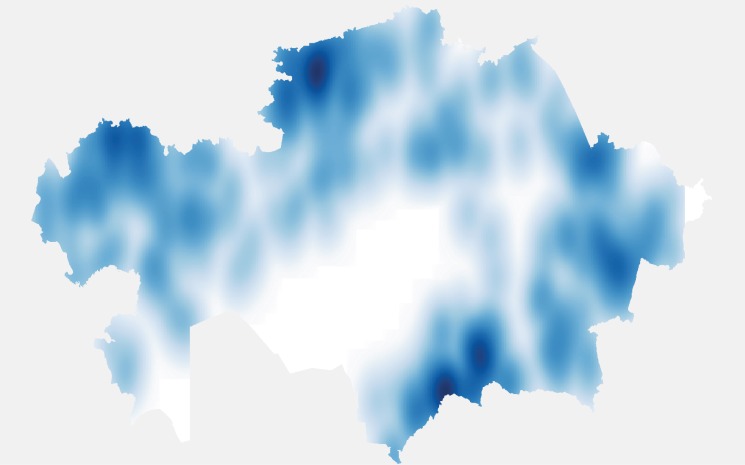
Kernel density estimation for outbreaks of animal rabies in Kazakhstan between 2003 and 2013. The darker the shading indicates a higher density of outbreaks. White is where there are no outbreaks.

**Fig 3:**
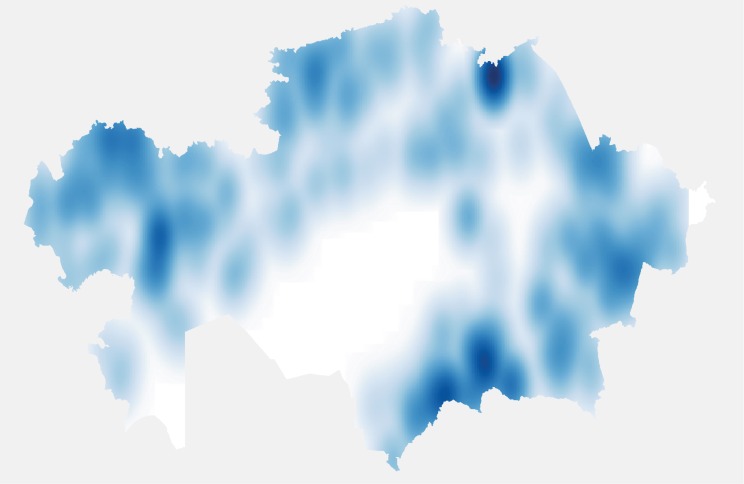
Kernel density estimation for cases of animal rabies in Kazakhstan between 2003 and 2013. The darker the shading indicates a higher density of rabies cases White is where there are no cases.

**Fig 4:**
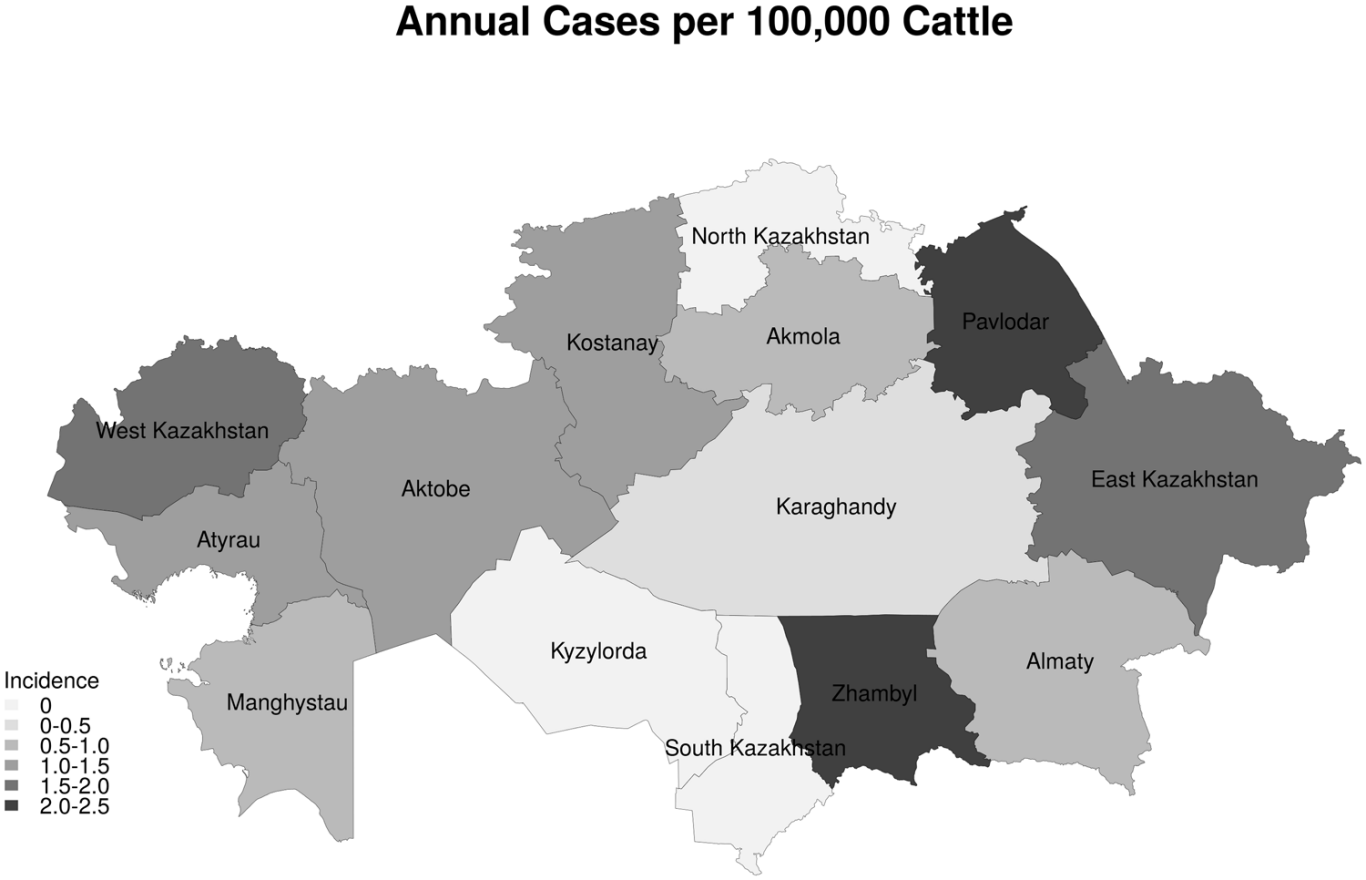
Annual incidence of rabies in cattle (confirmed cases per 100,000 cattle).

In total 1054 cases of animal rabies were reported between 2003 and 2013 in 778 outbreaks which had GIS coordinates. A further 350 cases in 2014 to 2015 in 306 outbreaks were reported only at the district level. Most outbreaks were single cases, but ranged up to a maximum of 64 cases in one outbreak. Cattle had the largest number of cases with 712 reported, followed by 242 cases in dogs, 226 cases in foxes, and 124 in sheep and goats (Fig 1). In total domestic farm animals accounted for 888 cases (62.8%), domestic carnivores (cats and dogs) 278 cases (19.7%), wild carnivores (foxes, wolves, jackals) 238 cases (16.8%) and other species 9 cases (0.6%). Rabies cases were reported from most geographical districts with the exception of Kyzylorda Oblast and parts of the west of Karaghandy Oblast. There were 2 distinct districts where there appeared to be a large clustering of outbreaks–in the south of Zhambyl Oblast and a second major cluster in the north of Kostanay Oblast. Other less intense clusters were seen in the north of West Kazakhstan Oblast and in East Kazakhstan Oblasts (Fig 2). In terms of actual cases, it would appear to be different with a further major cluster appearing in the north West of Pavlodar Oblast (Fig 3), but this is mainly due to a large single single outbreak involving 64 cattle. It was further possible to control for cattle population sizes. Fig 4 gives the annual incidence in cattle and confirms that Zhambyl Oblast has the highest incidence of cattle rabies at 2.4 cases per 100,000 cattle per year. High incidences were also seen in Pavlodar Oblast, West Kazakhstan Oblast and East Kazakhstan Oblast.

### Animal vaccination

**Fig 5:**
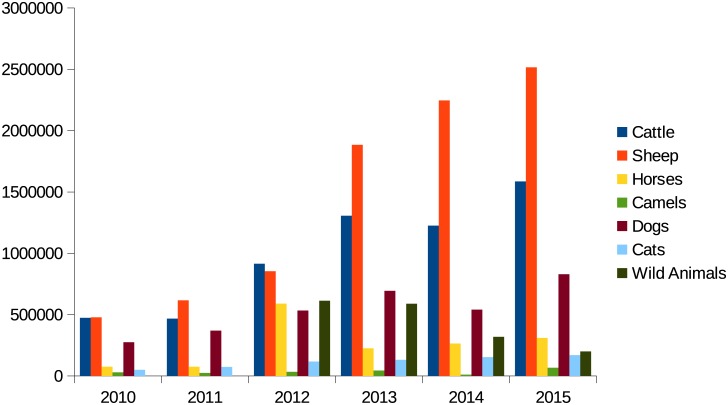
Numbers of animals vaccinated against rabies by species between from 2010 to 2015.

There has been a steady increase in the number of animals vaccinated. For example 473,000 cattle were vaccinated in 2010, rising to 1.6 million in 2015. In 2010 275,000 dogs were vaccinated, rising to 830,000 by 2015 (Fig 5).

A total of 18 pathological samples were collected from wild carnivorous in East Kazakhstan, West Kazakhstan, and Kostanay regions. Of these, 8 animals had consumed baits containing vaccine and tetracycline (44.4%, exact binomial 95% CIs 21.5%-69.3%).

The study results demonstrated that there were no antibodies precipitating rabies virus antigen in any of the blood serum samples collected from animals vaccinated by Raksharab. In blood serum samples of animals vaccinated by Russian Schelkovo-51 vaccine, antibodies in 0.32–0.6 ME/ml and 1–2 ME/ml have been identified, which demonstrates a modest level of immunity.

There were 216 cases of animal rabies registered in 2011, 139 in 2012, and 174 in 2013, 163 in 2014 and 187 in 2015 which indicates that there is little change in rabies incidence following widespread use of vaccination.

### Human rabies and DALY estimates

From 2010 to 2015 a total of 388,807 animal bite injuries were recorded. This equates to a bite incidence of 368/100,000 per year. Of these 386,785 were recommended for post exposure prophylaxis (PEP) with 385,733 completing the treatment (a mean of 64,289 per year). A small number refused PEP. There was a significantly increased risk of children between the ages of 6–14 years of age receiving a bite injury. There were 95,185 bite injuries in this age group. If they had received the number of bite injuries according to the population size of this age group, there should have been 50,501 (Chi Square, p<0.0001). Thus this group received 24.5% of all bite injuries, but represented only 13.0% of the total population.

Of the 388,807 animal bite injuries reported between 2010 to 2015, the lower extremities were the most likely to suffer a bite injury accounting for 49.7% of all injuries. The forearm, hand and fingers accounted for 28.5% of bite injuries, with the head, face and neck for 5.2% of injuries. The shoulders and trunk had 9.7% of injuries. Multiple injuries accounted for 3.7%. Subjects reported being licked by a suspect animal rather than a penetrating bite in 3.1% of cases.

There were a total of 50 human fatalities from 2009 to 2015 inclusive. This was an average of 7.1 cases per year. (Poisson 95% CIs 5.3–9.4) This ranged from a maximum of 14 seen in 2009 to a minimum of 3 seen in 2014. There was no evidence of any trend in the data or any significant differences in the number of cases recorded each year (Poisson regression, p>0.1). These rabies cases resulted in a disease burden of 457 DALYs (95% CIs 338–594) per year. This is the equivalent to approximately 64 DALYs per case of rabies. In total 385,773 bite injuries were recommended for PEP treatment. This resulted in approximately 1140 YLDs per year.

Of the 388,807 bite injuries, a diagnosis of rabies was confirmed in 3067 biting animals (0.78%). In the absence of PEP, this would have expected to result in an additional 713 (95% CIs 440–1121) cases of rabies or 118 (95% CIs 74–185) per annum. This would have resulted in 7657 DALYs (95% CIs 4723–12041) annually. With an estimated cost of PEP of $9.1 million per annum, the cost per rabies case averted is $76908 (95% CIs $34412-$166350), with the cost per DALY averted of $1193 (95% CIs $534-$2581). The observed number of cases was 36 from 2010 to 2015. It is not recorded if the cases of human rabies received any PEP, were members of the group who refused PEP or failed to seek any treatment following a bite. However, 1052 individuals over this period were bitten but refused to complete the recommended PEP. Assuming that they had a similar probability of being bitten by a rabid animal (0.78%), this would result in 8 people potentially exposed to the rabies virus but not undergoing treatment, with an expectation that perhaps 2 would have died of rabies. This indicates that at least 34 of the 36 cases of human rabies seen occurred in individuals who failed to seek any medical advice following their bite injury.

In total between 2010 and 2015 inclusive, 3820 dogs were investigated for rabies of which 242 were confirmed positive (6.3%). The total of other species investigated for rabies during this time period was 4492 of which 874 (19.5%) were confirmed as rabies. We therefore applied a mean probability of 0.063 to those individuals suffering dog bites and 0.195 to bite injuries from other species as the probability they were bitten by a rabid animal

Most bite injuries were caused by dogs (340907 of 388807 bite injuries or 87.7%) followed by cats (8.2%), cattle (1.1%), horses (0.44%), foxes (0.24%), wolves (0.07%) with the remainder unspecified. Assuming the transmission probabilities dependent on the anatomical location of the bite injury, this would have resulted in 1184 cases of rabies per annum (95% CIs 729–1869) or 76318 DALYs (95% CIs 47000–120470). In this scenario the cost per DALY averted is US$119 (95% CIs $52-$256), whilst the cost per case averted is US$7653 (95% CIs $3395-$16531)

Under this scenario, there is also a much higher probability that the 1052 individuals refusing PEP were actually bitten by a rabid animal. This would result in a mean of approximately 3 cases of rabies (95% CIs 2–5) or 207 DALYs (95% CIs 127–326) per annum. This is approximately half the observed number of human cases. In this scenario with the higher probability of the animal inflicting the bite having rabies it suggests that 19 people refusing PEP would go on to develop rabies and 17 individuals failed to seek medical advice.

### Costs

The total costs (direct and indirect) of seeking PEP treatment was estimated at $ 9.1million per annum (95% CIs $4.7 million- $16.6 million). Production losses from premature deaths were estimated as $5.4 million per annum (95% CIs $4.0 million- $7.1 million). A mean of $3.4 million per annum (range $2.5 million–$4.5 million) was allocated for the catching and destruction of stray and wild animals suspected to be affected by rabies. Vaccine was purchased at a cost of $0.35 per dose for use in livestock, dogs and cats. Oral baits containing vaccine for distribution to wildlife was purchased at $0.97 a dose. A mean of 4.7 million domestic animals per annum between 2013–2015 were vaccinated at a cost of $1.7 million per annum. Of this, $1.4 million was spend on vaccinating livestock with the remainder on dogs and cats. A mean of 736,000 baits per annum were distributed for wildlife at a cost of $719,000 per annum.

Livestock losses were relatively low, based on the value of livestock affected by rabies. Thus the losses attributed to cattle affected by rabies was US$ 20651 (95% CIs 18996–22442) per annum, sheep $627 (95% CIs $515-$755), camels $114 (95% CIs $59–193) and horses $2194 (95% CIs $1628-$2878). Total livestock losses were estimated as $23581 per annum (95% CIs $21818-$25512).

**Fig 6:**
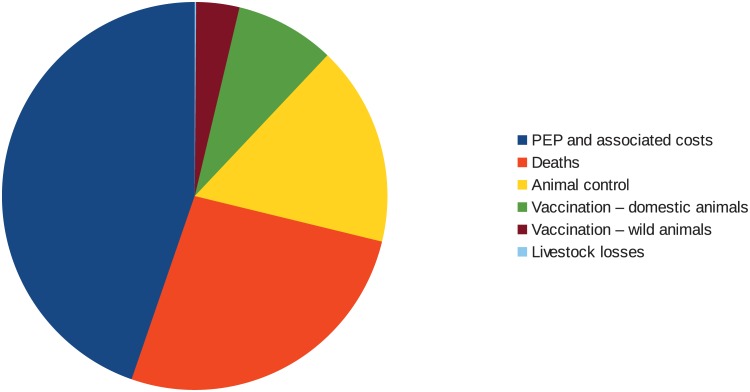
The relative contribution of different cost items to the total economic losses caused by rabies in Kazakhstan.

The total economic costs of rabies in Kazakhstan when adding up the costs from all sectors and the losses is approximately US$ 20.9 million per annum (95% CIs $15.7 million- $28.2 million). The relative contribution to these costs are illustrated in Fig 6.

### Regression analysis

The negative binomial GLM gave a highly significant association with the numbers of cases per oblast reported in agricultural animals and the numbers of confirmed diagnoses in foxes per oblast (Table 1). The relationship between the number of rabies diagnoses in dogs and agricultural animals was less highly significant with a smaller effect size. The numbers of cases in humans showed a significant relationship with numbers of cases diagnosed in dogs, but no relationship with cases in foxes.

**Table 1:** Results of GLM or regression of numbers of confirmed cases of humans or agricultural animals against the numbers of confirmed cases in fox and dogs by oblast.

Independent Variable	Number of Cases in Dogs		Number of cases in Foxes	
Dependent Variable	Parameter Value	P value	Parameter Value	P value
Number of Human Cases	0.03	0.048	ns	
Number of Cattle Cases	0.05	0.043	0.16	0.002
Number of Sheep Cases	ns		0.18	0.0002
Number of Horse Cases	0.05	0.01	0.11	0.007
Number of Camel Cases	ns		ns	
Total (Agricultural Animals)	0.04	0.049	0.15	0.0008

ns = not significant.

## Discussion

In Kazakhstan cattle appear to be over representative in the number of rabies cases, consisting of approximately 50% of total cases reported. In Mongolia cattle also appear to represent the majority of rabies cases, representing 80% of 1273 rabid animals reported between 1996 and 2004 [18]. However, livestock losses in total appear to be quite small and are much lower than the $1.2 million estimated in the GBR [1]. Our estimates of livestock losses are only laboratory confirmed cases and thus may be a substantial underestimate of the true extent of livestock losses. This may also be true of cases in wild and domestic carnivores. The regression analysis demonstrates a correlation between the number of cases in sheep, cattle and horses with the numbers of foxes rabies cases diagnosed. The statistical relationship with the numbers of cases in dogs was less marked. This is consistent with the hypothesis that foxes represent the principal reservoir and hence in Kazakhstan, sylvatic rabies is the main problem. Only camels failed to show this relationship, but this may be because of the very small numbers of camels confirmed as rabid and hence a sample size issue.

The detailed data which included the diagnosis in the animal responsible for the human bite injury enabled us to estimate the cost effectiveness of the PEP. This clearly demonstrated that PEP prevented a substantial number of rabies deaths. The cost at $1419 per DALY averted is cost effective in view of the annual GNI per head in Kazakhstan of approximately US$11860 (in 2014)[9]. Our data suggests that PEP prevented at least 713 rabies deaths over a 6 year period or a mean of 119 deaths per year. This is much smaller than the 4095 prevented deaths per annum in Kazakhstan suggested by the GBR study [1]. Our alternative scenario that the probability of receiving a bite injury from a rabid animal was equal to the proportion of animals that were laboratory diagnosed as rabies of those investigated gives a higher figure. In this scenario, there would be approximately 7106 rabies cases averted over the 6 year period or 1184 per year. This is closer to the GBR figure but is still substantially lower.

We also report a greater frequency of PEP treatment than the GBR study. Thus we report a bite incidence of 368/ 100,000 per year and 64,289 individuals completing PEP treatment per year. This compares to a bite incidence of 290/100,000 and 45,650 estimated by the GBR study [1]. However our confirmed PEP frequency is within the uncertainty limits of the GBR study. The fatalities of 7 per year are in agreement from both studies. It is not possible to speculate the degree of under reporting of human rabies cases. However, any fatal neurological disease is investigated and hence this would mitigate against under reporting. The concordance of our data reporting actual numbers of cases with that of GBR study which had estimates from models, would also be evidence that the degree of under reporting is not substantial.

The largest cost item was that of costs associated with PEP. This included the direct cost of vaccination, costs of travel and loss of income whilst seeking medical aid. These costs were estimated from the data in the GBR study as we were not able to access more reliable data for this study. The costs we estimated are a little higher than the GBR simply because our data demonstrated that there were greater numbers given PEP treatment than were estimated in the GBR study. Likewise we used the same methodology and data to estimate the losses due to premature mortality in those individuals who died of rabies. The main difference in our results was a narrower confidence limits as we were able to calculate the Poisson confidence intervals accurately from the numbers of cases reported in Kazakhstan.

The amount of money invested in animal control: i.e. controlling and destruction of stray and infected animals was $3.4 million per annum. There are increasing numbers of dogs associated with the livestock industry [19], increasing numbers kept as pets and for security as well as a substantial stray dog problem [20]. This sum is entirely allocated to the rabies control budget. However, human cystic echinococcosis (CE) is a major problem in Kazakhstan with a mean of 850 cases reported per annum between 2007 and 2013 [21]. Control of dogs is also undertaken for control of CE, thus is could be argued that some of this budget should be allocated to the control and surveillance of echinococcosis. The global burden of foodborne diseases suggested that each case of CE results in 0.97 DALYs [22], thus the burden of CE in Kazakhstan could be estimated at approximately 825 DALYs per annum. Therefore, if the animal control budget was allocated to these diseases according to the relative burden of human disease, then the $3.4 million would be allocated at a ratio of 457:825 resulting in $1.2 million allocated to rabies control and $2.2 million allocated to the control of echinococcosis. This would reduce the estimated economic impact of rabies.

The data indicate that despite the use of vaccination, there has been neither a reduction in the number of areas reporting rabies cases in farm animals nor a reduction in the total numbers of cases. This may indicate a lack of efficacy of the vaccination programme. One of the vaccines used appear to result in a poor antibody response and thus may be ineffective. The utility of vaccination in wild carnivorous was based on the results of uptake of the vaccine by such animals[23] provided by the Committee of Veterinary Control and Supervision of the Ministry of Agriculture, Republic of Kazakhstan. Following these results estimates were made for vaccination coverage for 2013. Over the endemic rabies foci, determined as 49,825 km^2,^, it was calculated that a total of 1,245,640 doses of the anti-rabies vaccine need to be disseminated in animal habitats.

Modifications of the vaccination programme may reduce the economic impact of rabies. Alternatively it might be argued that there is improving reporting of the disease in animals and hence the data might indicate a true decrease in the number of animal cases which is masked by the improved reporting. Vaccinating farm livestock does not prevent transmission and hence the only justifiable reason would be an economic gain from preventing livestock deaths due to rabies. With a cost of $0.35 per does of vaccine, the cost could be justified if the probability of being bitten by a rabid animal was sufficiently high such that the probability of animal death offset this cost. We have no data on the likelihood of farm animals being bitten and thus can not make this calculation. It is also arguable that it might be more cost effective to direct all resources to the vaccination of dogs and foxes. This could lead to elimination of the disease and would protect livestock livestock indirectly as there would no longer be a risk of exposure to rabid carnivores.

The number of individuals bitten by confirmed rabid animals was greater than the number of confirmed animal rabies cases. This clearly shows that rabid animals, usually dogs, will bite multiple times. Thus 2692 individual received bites from dogs confirmed infected with rabies, but there were only 242 confirmed rabies cases in dogs. Thus each rabid dog bit at least an average 10 individuals. This ratio is much higher than some other studies. In Chad in Africa there is a report of 23 dogs biting 37 individuals [24] and in Haiti it was reported that 53 dogs with confirmed rabies bit 57 people [25]. In contrast data from Poland is closer to the ratio we report. In 2012, 80 people received PEP following exposure to 13 dogs with confirmed rabies, i.e. on average 6 people were exposed to each rabid dog [26]. Likewise in 2011, 7 people were exposed on average to each rabid dog [27]. This might be contextual dependent with middle and upper income countries reporting greater incidences of dog bites with low income countries under reporting such injuries. However, major clinical signs in dogs are aggression, biting, laryngeal paralysis, mastication muscle paralysis, roaming, abnormal barking, and excessive salivation. Dogs often bite when mildly provoked [28]. Thus it would not be surprising that a dog with clinical rabies may bite a number of people.

### Conclusion

The results of this study give a detailed overview of the distribution and burden of rabies in Kazakhstan. The results are largely based on detailed surveillance and budgetary data provided by the veterinary and public health services in Kazakhstan, although some data used in the estimates were borrowed from the GBR study. This study also demonstrates that rabies has a substantial economic effect in Kazakhstan. Direct measures to prevent the disease in humans, notably PEP appear to be cost effective, even though PEP itself does not prevent transmission. Other measures such as widespread vaccination of livestock appear to be less cost effective with near $1.4 million invested in vaccinating farm livestock with no evidence of a change in animal incidence. Thus these resources might be better targeted towards greater vaccination of the reservoir hosts to prevent transmission

## Supporting Information

S1 Dataset R code and data sets. This folder contains all the data used in the manuscript. The file also includes the R code used to estimate the burden of disease, economic losses and regional incidences. Relevant shape files used to generate the maps and associated R code are also provided.
(ZIP)
