# Supplementary material for: Rabies in Kazakhstan
Source: PLoS Negl Trop Dis. 2016 Aug 3;10(8):e0004889. doi: 10.1371/journal.pntd.0004889 (PMC4972401; doi:10.1371/journal.pntd.0004889)
Supplement: S1 Dataset — This folder contains all the data used in the manuscript. The file also includes the R code used to estimate the burden of disease, economic losses and regional incidences. Relevant shape files used to generate the maps and associated R code are also provided. (ZIP) [file pntd.0004889.s001.zip › supplementary data/Rcodeandfiles/Rcode.doc]

```
R Code

#Plot of Cattle Incidence:

library("maptools")
cxx <- readShapePoly("KAZ_adm1.shp",proj4string=CRS("+proj=longlat"))
colours <- c("white","grey92","grey78","grey67", "grey50","black")
co=c(0,0.01, 0.5, 1.0,  1.5,  2.0, 2.5, 3000)
plot(cxx,col=colours[findInterval(cxx$CASES, co)])
box=c("0","0-0.5","0.5-1.0","1.0-1.5","1.5-2.0","2.0-2.5")
legend(44.5, 45, legend=box, border="black", bty="n",fill=colours, y.intersp=.75, title="Incidence", cex=0.9)
text(getSpPPolygonsLabptSlots(cxx), labels=cxx$NAME_1, cex=0.6)
title(main="Annual Cases per 100,000 cattle", cex.main=1.5)


#Kernal Density Plots of Rabies Outbreaks

Library(graphics)

#First a  smoothed colour density representation of the scatterplot, obtained through a kernel #density estimate
rabies<-read.csv(“rabies.csv”, header=T)
smoothScatter(x=rabies$lon_dd, y=rabies$lat_dd, xlim=c(40,95), ylim=c(40,56), nrpoints=0, xlab="", ylab="", axes=F)


# Load up cordinates of an outline map of kazakhstan
coor1 <- read.csv(coor1.csv)


#Overlays outline kazakhstan map on the smoothed scatter plot
xrange<-range(coor1$long, na.rm=TRUE)
View(xrange)
yrange<-range(coor1$lat, na.rm=TRUE)
xbox<-xrange+c(-2,2)
ybox<-yrange+c(-2,2)
polypath(c(coor1$long, NA, c(xbox, rev(xbox))),
                   c(coor1$lat, NA, rep(ybox, each=2)),
                 col="light grey", rule="evenodd", border=NA)


#DALYs and Financial Losses
#Generate data set of 1000 cases at a rate of 50 every 7 years (ie 7.1 per year)
cases<-rgamma(10000, 50, 7)
#Observed bites in each age group, probabilities of bite, mid age of each group, residual life expectancy
obsbites<-c(39535, 95185, 23833, 41843, 29853, 27554, 26514, 24445, 80045)
propbites<-obsbites/388807
ages<-c(2.5, 10, 16, 21.5, 29, 33, 38, 43, 59)
lifexp<-c(89.51, 82.03, 70.57, 76.04, 63.11, 59.13, 54.18, 49.24, 33.62)
YLLcase<-propbites*lifexp
cases2<-as.matrix(cases)
probs<-cases2%*%YLLcase
probs2<-rowSums(probs)
quantile(probs2, prob=c(0.025,0.5,0.975)) #median YLLs and CIs (=DALYs as fatal)
phead<-rbeta(10000, 7.159, 5.858) # prob rabies from head bite
puextr<-rbeta(10000, 7.74, 27.45) #prob rabies from upper extremity bite
ptrunk<-rbeta(10000, 0.586, 5.925)
plext<-rbeta(10000, 5.918, 43.4)
anbites<-c(20400,49677,125329,193401)


untreated<-rbeta(10000, 3068, 385741) #proportion untreated
headbites<-rbeta(10000, 20401,368406) #proportion head bites
rabhead<-untreated*headbites*388807*phead
probdogspos<-rbeta(10000, 237, 3584)
probother<-rbeta(10000, 875, 3518)
uexbites<-rbeta(10000, 40677, 34131)
trbites <-rbeta(10000, 125329, 263479)
lexbites<-rbeta(10000, 193401, 195407)
rabuex<-untreated*uexbites*388807*puextr
rabtrunk<-untreated*trbites*388807*ptrunk
rablex<-untreated*lexbites*388807*plext
rabtotal<-rabhead+rabtrunk+rabuex+rablex
rabtotal1<-as.matrix(rabtotal)
probs3<-rabtotal1%*%YLLcase
probs4<-rowSums(probs3)
quantile(probs4, probs=c(0.025, 0.5, 0.975))/6 #DALYs per year averted-scenario 1

#cattle losses
catcases<-rgamma(10000, 563, 6) # number of cases per year
catpop<-rgamma(10000, 35428000,6) # number of cattle in KAZ
propcatrab<-catcases/catpop #proportion with rabies
catlosses<-propcatrab*1300000000 # proportion with rabies * value of cattle production
quantile(catlosses, probs=c(0.025, 0.5, 0.975)) #loss of cattle production

#camel losses
camcases<-rgamma(10000, 12, 6) #number of cases per year
campop<-rgamma(10000, 824000, 5)#camel pop in KAZ
propcamrab<-camcases/campop
camlosses<-propcamrab*9620000
quantile(camlosses, probs=c(0.025,0.5,0.975))

#horses losses
horcases<-rgamma(10000, 47,6)
horpop<-rgamma(10000, 3645907,2)
prophorrab<-horcases/horpop
horlosses<-prophorrab*514000000
quantile(horlosses, probs=c(0.025,0.5,0.975))

#sheep losses
shpcases<-rgamma(10000, 105, 6)
shppop<-rgamma(10000,104032800, 6)
propshprab<-shpcases/shppop
shplosses<-propshprab*622000000
quantile(shplosses, probs=c(0.025,0.5,0.975))

#agricultural animal losses
agriloss<-catlosses+camlosses+shplosses+horlosses
quantile(agriloss, probs=c(0.025,0.5,0.975) #losses to agricultural animals

#money into control of strays etc
straycosts<-rnorm(10000,3545120.43395671, 263312.22685604)


#Calculating DALYs averted
rabhead1<-(probdogspos*headbites*340907*phead)+(probother*headbites*47900*phead)
rabuex1<-(probdogspos*uexbites*340907*puextr)+(probother*uexbites*47900*puextr)
rabtrunk1<-(probdogspos*trbites*340907*ptrunk)+(probother*trbites*47900*ptrunk)
rablex1<-(probdogspos*lexbites*340907*plext)+(probother*lexbites*47900*plext)

rabtotal1<-rabhead1+rabtrunk1+rabuex1+rablex1
quantile(rabtotal1, probs=c(0.025, 0.5, 0.975))/6 #Rabies cases per annum averted
rabtotal2<-as.matrix(rabtotal1)
probs3<-rabtotal2%*%YLLcase
probs4<-rowSums(probs3)
quantile(probs4, probs=c(0.025, 0.5, 0.975))/6 #DALYs per year averted-high scenario


#Number of cases by species in each Oblast from 2010-2015
dogs<-c(12,22,2,4,26,70,15,3,28,1,2,6,3,40,0,2)
humans<-c(0,2,2,1,1,3,2,1,1,7,0,1,0,15,0,0)
cattle<-c(40,36,54,24,209,74,60,2,38,0,0,21,0,5,0,0)
fox<-c(3,5,9,5,26,12,20,0,9,2,2,14,6,3,0,1)
sheep<-c(0,4,3,14,25,18,8,0,1,0,0,29,0,3,0,0)
camel<-c(0,7,0,1,0,0,0,0,1,0,4,0,0,0,0,0)
horse<-c(5,0,4,1,18,11,0,1,4,0,1,1,0,1,0,0)
livestock<-cattle+sheep+camel+horse

library(MASS)

#Regression of number of cattle cases with fox cases
result1<-glm.nb(cattle~1)
result1<-glm.nb(cattle~fox, init.theta=results1$theta)
summary(result1)


#Regression of number of human cases with dog cases
result1<-glm.nb(human~1)
result1<-glm.nb(human~dogs, init.theta=results1$theta)
summary(result1)

#Regression of number of livestock cases with fox cases
result1<-glm.nb(livestock~1)
result1<-glm.nb(livestock~foxes, init.theta=results1$theta)
summary(result1)


#Fitting Uncertainty from GBR data and estimating total costs

rBeta_ab(10, shape1 = pars$alpha, shape2 = pars$beta, a = pars$a, b = pars$b)
library(prevalence)
library(ExtDist)
pars1<-betaPERT(0.92, 1.41, 6.3)# A median of 1.9 of with CIs of 0.99-4.3 (costs of PEP x 106 in GBR)
pars2<-betaPERT(21500, 33300, 146000)# A median of 45620 with Cis (95% CIs 23,421 to 99,821)
costPEP<-rBeta_ab(10000, shape1=pars1$alpha, shape2=pars1$beta,  a = pars1$a, b = pars1$b)
nosPEP<-rBeta_ab(10000, shape1=pars2$alpha, shape2=pars2$beta,  a = pars2$a, b = pars2$b)
PEP<-1000000*costPEP/nosPEP
quantile(PEP, c(0.025, 0.5,0.975) #cost per treatment of PEP
pars3<-betaPERT(2.6,4.05,17.8) #lost income whilst treated
lostinc<-1000000*rBeta_ab(10000, shape1=pars3$alpha, shape2=pars3$beta,  a = pars3$a, b = pars3$b)
pars4<-betaPERT(125000,132000,1500000, k=60)#travel costs
losttrav<-rBeta_ab(10000,  shape1=pars4$alpha, shape2=pars4$beta,  a = pars4$a, b = pars4$b)
PEPtot<-PEP*64289 #total cost of PEP per year
costdeath<-probs2*11860 #cost of fatal cases
costvac<-0.35*rnorm(10000,4744310,372141) #cost vaccinating livestock
costvac1<-0.97*rnorm(10000,736403,292736) #costs vaccinating wildlife
totalcosts<-agriloss+PEPtot+costdeath+straycosts+lostinc+losttrav+costvac+costvac1
quantile(totalcosts, c(0.025, 0.5, 0.975))

quantile(((PEPtot+lostinc+losttrav)*6/rabtotal1), c(0.025,0.5,0.975)) #cost per rabies case averted
quantile(((PEPtot+lostinc+losttrav)*6/probs4), c(0.025,0.5,0.975)) #cost per DALY averted
```
